# Factors Influencing Customer Satisfaction and Behavioral Intention in Local Food Tourism: A Structural Equation Modeling Approach

**DOI:** 10.12688/f1000research.179249.5

**Published:** 2026-08-21

**Authors:** Warach Madhyamapurush

**Affiliations:** 1 School of Business and Communication Arts, University of Phayao, Thailand 19, Mueang Phayao District, Phayao, 56000, Thailand

**Keywords:** Local food tourism, customer satisfaction, behavioral intention, recommendation intention, food quality, cultural experience, structural equation modeling.

## Abstract

Local food tourism (LFT) significantly impacts destination experiences by shaping tourists’ perceptions, satisfaction, and post-visit behavior. Despite the growing importance of local food tourism, there remains limited empirical understanding of how experiential attributes influence customer satisfaction and behavioral intention (BI). This research uses a thorough Structural Equation Modeling (SEM) framework to examine the variables affecting customer satisfaction (CS) and behavioral intention (BI) in LFT. A total of 380 valid responses were obtained from a quantitative cross-sectional survey of visitors who sampled local cuisine at particular Thai culinary sites. A structured questionnaire measured six exogenous constructs: food quality (FQ), Authenticity (AU), service quality (SQ), physical environment (PE), cultural experience (CE), and perceived value (PV) along with CS as a mediator and BI (revisit intention and Consumer referral behavior) as the endogenous outcome. Cronbach’s alpha, composite reliability (CR), average variance extracted (AVE), and discriminant validity using the Fornell-Larcker criterion were used to evaluate reliability and validity. Utilizing confirmatory factor analysis (CFA), the measurement model was validated. The findings suggest that CS is strongly and favorably influenced by FQ, AU, CE, and PV. The association between experience qualities and loyalty outcomes is partially mediated by CS, which also strongly predicts BI. A well-fitting measurement model is indicated by the model’s acceptable goodness-of-fit indices, which include a Comparative Fit Index (CFI) of 0.952, Tucker–Lewis Index (TLI) of 0.945, Root Mean Square Error of Approximation (RMSEA) of 0.056, and Standardized Root Mean Square Residual (SRMR) of 0.048. The findings provide practical insights for enhancing tourist loyalty through authentic and high-quality culinary experiences.

## 1. Introduction

Tourism is evolving as contemporary travelers increasingly seek immersive experiences that extend beyond traditional sightseeing. Rather than passively observing destinations, tourists now desire meaningful engagement with local culture, traditions, and everyday life. Food has taken center stage and is giving sensory, emotional and cultural satisfaction. Food experiences enable the visitors to visit destinations through meaningful ways. ^1^ LFT is a tourism where one goes to a particular place to have an experience of its gastronomy culture and traditional cuisine. It is a combination of food and culture that can be remembered. To the tourists, local food is a part of the identity and heritage of a destination, it is important in determining the entire travel experience. ^2^ The quality of the cuisine is a major factor in determining how satisfied tourists are with their gastronomic experiences. The freshness, flavor, presentation, and AU of a destination’s cuisine all influence how people perceive it; delicious food elevates enjoyment and positive sentiments about a place. For this reason, FQ is among the first things that local businesses and tourism managers are interested in. ^3^ A very important factor in making food tourism experiences memorable is AU. Tourists are interested in foods that contain some of the traditional ways of cooking and local cuisines. True food experiences enhance cultural affiliations and offer the feeling of distinctiveness. AU is another aspect that is usually looked at by tourists as a representation of heritage and uniqueness of a destination. ^4^ A critical debate in LFT revolves around the tension between authenticity and commercialization. While authenticity is often perceived as the core value of culinary tourism, reflecting traditional preparation methods, local ingredients, and cultural heritage, increasing commercialization driven by tourism demand may lead to the standardization and modification of local cuisines to suit global tastes. This creates a paradox where efforts to make local food more accessible and profitable can dilute its cultural integrity. Some scholars argue that staged authenticity may still provide meaningful experiences to tourists, whereas others emphasize that excessive commercialization risks undermining the originality and heritage value of local gastronomy. Therefore, understanding this dynamic is essential for balancing economic benefits with cultural preservation in LFT contexts.

Tourists’ dining experiences and degree of happiness are significantly impacted by the quality of the service. Visitors’ perceptions of a destination are enhanced by thoughtful, polite, and efficient treatment. Poor service is a major component of the entire culinary experience and can negatively affect satisfaction even with good FQ. ^5^ Customers’ overall enjoyment and satisfaction are greatly influenced by a restaurant’s physical environment, which includes its ambiance, seating arrangements, cleanliness, and location. A pleasant and attractive setting adds PV to the meal and improves the whole dining experience. ^6^ LFT plays a role in terms of CE as it helps visitors to connect local cultural practices, festivals and food. Some of the ways through which the tourists can interact with the culture are cooking demonstrations, local markets, and traditional events. These experiences make the interactions memorable and not exactly to taste food. ^7^ PV influences consumer attitudes that tourists develop towards culinary experiences in relation to cost and effort. Tourists ask themselves whether the experience is worth the money, time, and effort spent. Perceived high value reinforces satisfaction and increases the chances of positive behavior intentions. It connects the physical and the spiritual side of the experience. ^8^ CS is a key variable in tourism, which determines visitor behavior. To a larger extent, satisfied tourists revisit the destinations and idolize the experiences to other people. There are various factors which influence satisfaction and these factors are FQ, AU, service, environment, CE and PV. Knowledge of it assists managers to improve the overall tourism experiences. ^9^ BI is the likelihood that travelers will return to or recommend a place. In tourist research, it is a typical indicator of loyalty, and positive experiences and high levels of satisfaction increase the likelihood that a person will return or actively advocate a place. These actions are essential for the tourism industry’s sustainable growth. ^10^ The relationship between satisfaction and BI is quite strong in tourism literature. Content customers increase their repeat visits, make referrals. The occurrence of genuine food, attentive services and involvement in interesting cultural activities raises the chances of such an action, and it underlines the significance of a holistic concept about culinary tourism. ^11^ Tourists’ perceptions of local dining experiences are influenced by a variety of factors, including sensory quality, service, CEs, and the whole environment. Positive experiences in these areas increase customer satisfaction and strengthen behavioral intention. When determining the overall tourism experience, all of the factors work in tandem with one another. ^12^ Enhancing visitor happiness and encouraging return visits are the practical implications of this research for tourism stakeholders. Managers and local businesses can devise measures to enhance experiences, deliver expectations of the visitors, and enhance destination attractiveness. Offering cultural immersion, good quality, and delightful food experiences contribute to the long-term loyalty. ^13^ LFT provides a different approach of involving the visitors and advertising destination identity. Satisfaction and loyalty behaviors are influenced by the different elements of FQ and AU, service, environment, CEs, and PV. These aspects are important to understand to enhance tourist experiences. Culinary tourism is one of the crucial fields of developing memorable and sustainable tourism products. ^14^ Experience-based features like FQ, destination image, and experiential value have become the focus of the LFT research in explaining the behavior of tourists. These dimensions are significant to the CS and behavioral outcomes. An extensive model that incorporates several experiential features give more in-depth understanding of the overall tourism experience. ^15^ Figure 1 highlights the roles of FQ, AU, SQ, and other elements in shaping customer perceptions and future behavior.

**Figure 1.**
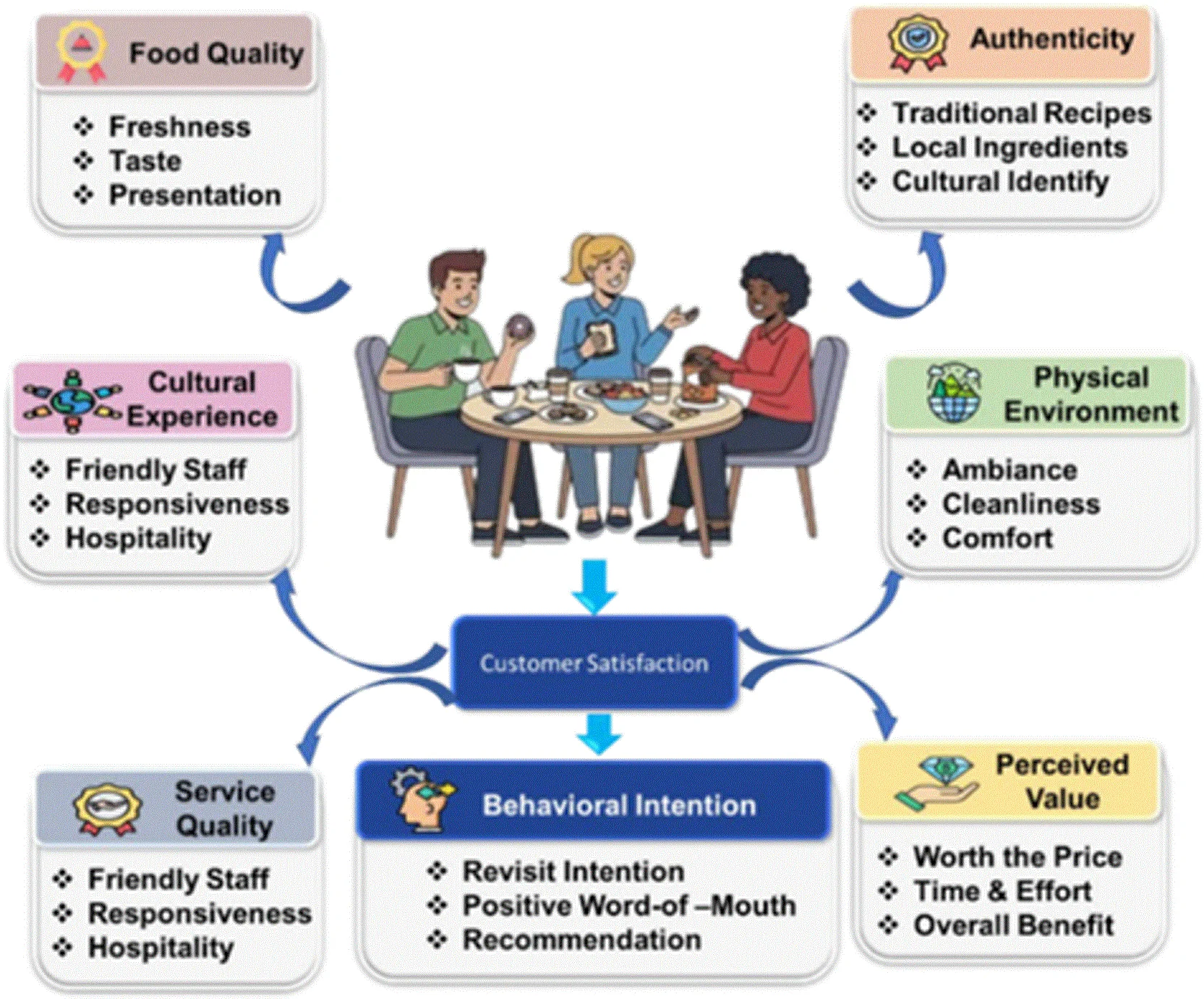
Determinants of CS in LFT.

The increasing significance of LFT makes it clear that there is a necessity to investigate the role of numerous experiential determinants in the CS level and the BI. Nevertheless, the two factors are normally analyzed separately, restricting the understanding of the overall impact of the two. Research seeks to bring about various experiential qualities, which include SQ, PE, FQ, AU, and CE, into one model with the aim of increasing tourist satisfaction, loyalty, and sustainable development in the LFT.

• The research examines the effect of FQ, SQ, AU, PE and the perception of value on CS and the BI in the LFT environments.
• To gather primary data on the key constructs, FQ, AU, quality of service, PE, PV, CS, and BI, a quantitative survey of 380 tourists who tried local cuisine in the chosen culinary destinations was provided.
• The research implements tests of reliability and validity such as Cronbach’s alpha, CR, AVE, and discriminant validity, and subsequently CFA and SEM are used to test direct, mediated and moderated relationships leading to a validated model of drivers of tourist satisfaction and loyalty outcomes in LFT.

The research is structured into seven parts. The introduction to the importance of LFT and its effects on customer satisfaction and BI is presented in Section 1. Section 2 is a literature review of pertinent literature on FQ, AU, SQ and other aspects of experience in LFT. Section 3 gives the framework of the hypotheses, which outlines the relationships between the major constructs. Section 4 describes the methodology, such as the data collection and measurement instruments. In Section 5, results of the SEM analysis presented. The discussion is examined in Section 6 and provide practical implications to the tourism managers. Section 7 is the last section, which ends with future research recommendation on LFT.

Recent scholarship highlights a growing tension in local food tourism between authenticity preservation and commercial standardization, where destinations often commodify traditional cuisine to meet mass tourism demand while risking cultural dilution. ^3, 14^ This debate emphasizes that while authenticity enhances experiential value and emotional connection, excessive commercialization may undermine perceived genuineness and long-term destination loyalty. Contemporary studies further argue that integrating experiential quality with cultural sustainability is essential for maintaining competitive advantage in LFT. ^2, 28^ Therefore, a more nuanced and integrative analytical approach is required to understand how multiple experiential constructs simultaneously influence satisfaction and behavioral intention within this evolving tourism landscape.

## 2. Literature review

Earlier empirical research on LFT and its satisfaction, and BI have discussed numerous variables such as the quality of food, SQ, AU, and CE. Nevertheless, some of the studies are constrained by small sample sizes, being destination specific, and cross-sectional thus limiting the generalizability and temporal applicability. The objectives of the research, the characteristics of the sample, the methodologies, the main findings, and the limitations of these researches are summarized in Table 1. It presents uniform results that FQ and AU have a positive effect on satisfaction and BI. Nevertheless, no exhaustive models that incorporate all these factors have been developed and thus the gap that this research seeks to fill in is to provide a more holistic understanding.

**Table 1.** Empirical studies on LFT and BI.

Ref	Objective	Sample	Method	Key findings	Limitations
^16^	Examine Theory of Planned Behavior (TPB) variables influencing international tourists’ local food consumption intention	457 tourists	Partial Least Squares SEM **(**PLS-SEM)	Attitude significantly predicted intention; responsible behavior moderated effects	Single destination; cross-sectional
^17^	Investigate food consumption values and intention with neophobia/neophilia moderation	250 respondents	SEM	Consumption values influenced attitude; neophilia (+) and neophobia (−) affected intention	Regional sampling; cross-sectional
^18^	Explore destination food image, neophobia, and BI	292 tourists	PLS-SEM	Food image and neophobia significantly shaped intention	Single country; cross-sectional
^19^	Examine heritage food tourists’ intention and destination image	336 tourists	PLS-SEM	Experiential value enhanced attitude, image, and intention	Cross-sectional design
^20^	Assess food culture attributes affecting satisfaction and patronage intention	172 attendees	SEM	Food culture improved satisfaction; satisfaction mediated patronage intention	Convenience sampling; small sample
^21^	Analyze determinants of revisit and recommendation intention (Extended TPB)	4,268 tourists	SEM	Quality and value enhanced satisfaction; satisfaction drove revisit/recommendation	Single destination context
^22^	Examine satisfaction and revisit intention toward local food heritage	62 respondents	Survey analysis	Price strongest satisfaction factor	Very small sample; self-reported data
^23^	Analyze determinants of satisfaction and revisit intention	200 respondents	SEM	PE influenced satisfaction; satisfaction predicted revisit	Online cross-sectional data
^24^	Examine FQ perception, satisfaction, and BI	487 tourists	Structural modeling	Core food & SQ enhanced satisfaction; satisfaction increased intention	Single destination
^25^	Study food experiences, attitude, image, and revisit intention (TPB-based)	526 tourists	SEM	Food experience improved attitude and image; mediated revisit intention	Convenience sampling
^26^	Assess gastronomy tourism quality and loyalty intentions	462 tourists	SEM-PLS	Gastronomy quality enhanced satisfaction; satisfaction mediated loyalty	Single city focus
^27^	Examine culinary experience quality and destination satisfaction	401 tourists	SEM	FQ strongest predictor of satisfaction and intention	Domestic sample; cross-sectional
^28^	Analyze food experience value, image, and revisit intention	458 respondents	SEM	Experience values influenced image and revisit intention	Single brand case
^29^	Analyze your feelings, place attachment, meal experience, and intention to return	408 tourists	SEM	Emotion and attachment were improved by eating, and the inclination to return was raised	Single destination
^30^	Examine gastronomic experience and revisit intention with mediation of satisfaction	525 visitors	Quantitative analysis	Esthetic experience influenced revisit; satisfaction mediated effects	Convenience sampling
^31^	Impact of 7Ps and service factors on satisfaction	400 hotel customers (Thailand)	Survey & regression	Most factors significant; distribution not	Region-specific; self-reported data
^32^	Examine evolution of culinary tourism (2001–2025)	Literature across phases	Critical review	Shift to authenticity, digital influence, sustainability gap	No primary data, limited regional depth
^33^	Examine sustainability impact of food tourism	Key stakeholders	In-depth interviews	Supports economy, culture, environment	Cost, over-tourism, authenticity issues

Existing research on LFT that integrate various experiential factors influencing CS and BI are limited. Although FQ, AU, SQ, and CE have all been studied separately, little study has been done to integrate these dimensions into a single model. By combining FQ, CE, AU, SQ PE, and PV into a thorough model to investigate their combined effects on visitor satisfaction and BI, this research closes this gap. The suggested model offers insightful information for improving visitor experiences and encouraging loyalty in LFT.

The present study has three major differences from previous SEM-related tourism studies. First, most previous research has focused on one or two experiential predictors, such as food quality or authenticity alone. The limitation here is conceptual rather than merely one of coverage. A diner does not evaluate freshness, service, atmosphere and cultural meaning as separate judgements and then add them up; these elements are encountered together in a single sitting, and each one conditions how the others are read. A single-predictor model therefore cannot represent the foodscape as the traveller actually meets it, and it is particularly ill-suited to the post-pandemic traveller, for whom a meal is weighed as an experience worth the journey rather than as a service worth its price. Capturing that requires the predictors to be estimated simultaneously, which is what this study does by combining six experiential constructs (FQ, AU, SQ, PE, PV, and CE) in one structural model. Second, this study differs from those of Angelakis et al. (2023) ^21^ and Sangkaew et al. (2025), ^13^ which only consider satisfaction as a mediator between service-related attributes and satisfaction, by treating PV as a partial mediator between service-related attributes and satisfaction, and CE as a cultural moderator of the relationship between satisfaction and intention. Third, this study goes beyond Western culinary tourism paradigms by examining the context of Thai LFT, which is unique in terms of cultural heritage preservation and commercialization pressures. The research gap is thus well defined: there is no integrative model that tests the cognitive, affective, and cultural mechanisms that influence tourist loyalty in LFT.

## 3. Research methods

The theoretical foundation of the hypotheses proposes that FQ, AU, SQ, and PE serve as key experiential drivers that influence CS and PV, which in turn determine tourists’ BI in local food tourism. PV is proposed as a mediating variable between SQ and PE and CS, meaning that SQ and PE influence satisfaction indirectly through their impact on perceived value. Table 2 demonstrates CE is suggested as a moderating variable that enhances the association between CS and BI, meaning that a high level of cultural immersion increases the impact of satisfaction on behavioral intention.

**Table 2.** Summary of hypotheses and proposed relationships in the conceptual model.

Hypothesis	Statement	Relationship	Explanation
**H1**	Food Quality (FQ) → Customer Satisfaction (CS)	Positive & Significant	Freshness, taste, presentation, and nutritional value enhance tourists’ dining experience, leading to higher satisfaction when expectations are met or exceeded.
**H2**	Authenticity (AU) → Customer Satisfaction (CS)	Positive & Significant	Authentic food creates cultural connection and immersion, improving overall experience and increasing satisfaction.
**H3**	Service Quality (SQ) → Perceived Value (PV)	Positive & Significant	Responsiveness, professionalism, and empathy improve service experience, increasing perceived worth of time and money.
**H4**	Physical Environment (PE) → Perceived Value (PV)	Positive & Significant	Cleanliness, ambiance, design, and comfort enhance the dining setting, leading to higher perceived value.
**H5**	Perceived Value (PV) → Customer Satisfaction (CS)	Positive & Significant	When tourists perceive benefits outweigh costs, their satisfaction increases.
**H6**	Customer Satisfaction (CS) → Behavioral Intention (BI)	Positive & Significant	Satisfied tourists are more likely to revisit and recommend, strengthening loyalty intentions.
**H7**	SQ → PV → CS	Mediation Effect	Service quality improves perceived value, which in turn enhances customer satisfaction.
**H8**	Customer Engagement (CE) moderates CS → BI	Moderation Effect	The strength of the relationship between satisfaction and behavioral intention depends on the level of customer engagement.

The more a tourist has a high cultural immersion, the more likely their satisfaction is to translate into BI such as returning to or recommending the destination. In other words, CE strengthens the effect of satisfaction on BI. Figure 2 represents the hypothesized relationships among FQ, AU, CE, SQ, PE, and PV in LFT.

**Figure 2.**
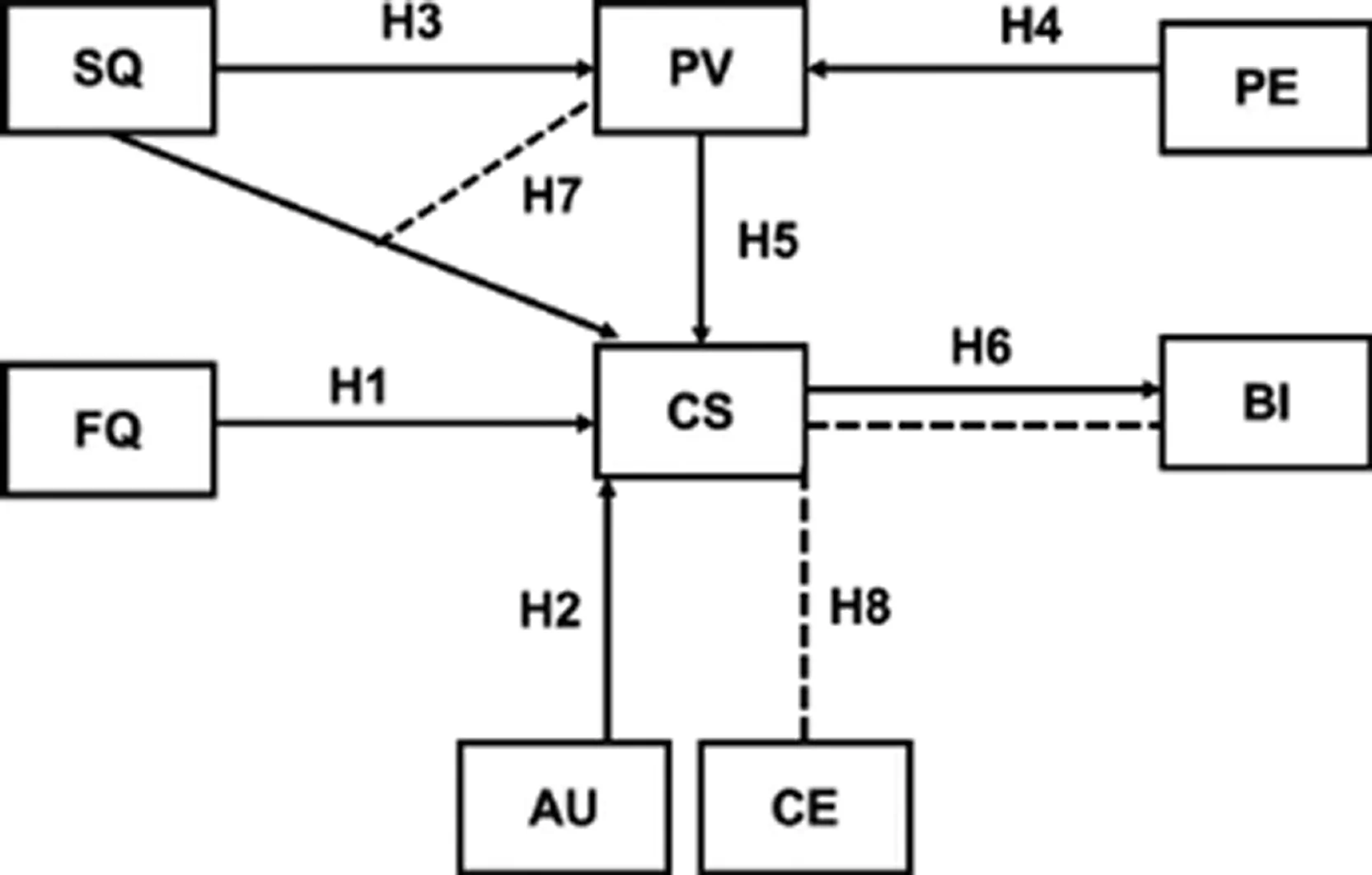
Structural model of influences on satisfaction and BI in food tourism.

The research adopted a quantitative research design and the surveyed data (real experiences of 380 tourists who have taste local food in the identified culinary destinations in Thailand) was modeled using statistical modeling tools. Experience features that the research analyzed FQ, AU, SQ, PE, and PV and their impacts on CS and BI. Measurement of all constructs was done through validated Likert-scale items and testing of structural models was through SEM. The suitability of the framework was evaluated based on reliability, validity and multicollinearity tests. Figure 3 shows the research methodology flow, from hypothesis framework to result evaluation.

**Figure 3.**
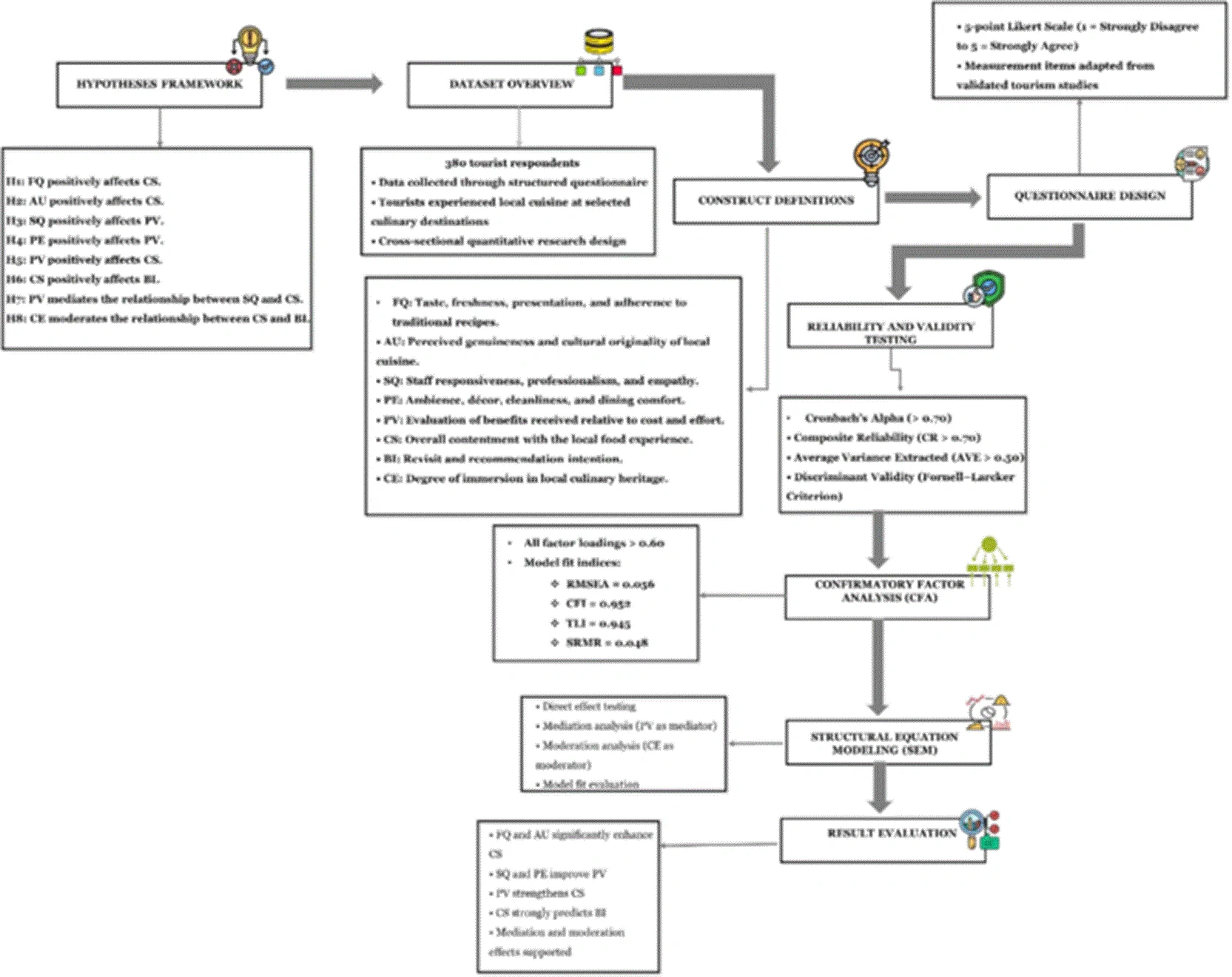
Methodology flowchart for assessing factors in LFT.

### 3.1 Research methodology and analytical framework

Research gathered 380 valid answers of tourists in Thailand who tested the local food in the chosen food outlets. The measurement of eight latent constructs was conducted by using a structured questionnaire according to the validated scales; FQ, AU, SQ, PE, PV, CS, BI, and CE. ^31^ The questionnaire underwent pre-test to ensure the questionnaire was understandable, reliable, and content valid. The data were obtained both online and offline to have accuracy and representativeness. The obtained dataset was a good empirical foundation to test the hypothesized connections and perform structural model analysis.

The key variables in this research and their definitions are presented in Table 3. The table also indicates the role of each variable as independent, mediator, moderator, or dependent in the research.

**Table 3.** Variables and their definitions.

Variable	Definition
FQ	The sensory, nutritional, and overall appeal of local cuisine, including taste, freshness, presentation, and adherence to traditional recipes. Acts as an independent variable.
AU	The perceived genuineness and traditional nature of the local food experience, reflecting local ingredients, cooking methods, and culinary heritage. Independent variable.
SQ	The responsiveness, professionalism, empathy, and efficiency of staff at culinary destinations. Independent variable.
PE	The dining setting, including ambience, décor, cleanliness, seating comfort, and overall aesthetics. Independent variable.
PV	The overall evaluation of benefits received relative to cost and effort invested. Mediates the relationship between SQ, PE, and CS.
CS	Overall satisfaction of visitors with their local dining experience. Mediates the connection between BI, PV, and experiential characteristics.
BI	The possibility that visitors will return or suggest the location.
CE	The extent of tourists’ immersion in the local culinary culture and heritage. Moderates the relationship between CS and BI.

The research adopted the available constructs and measurement items to test the relationships of experiential attributes, PV, CS, CE and behavioral intent. All variables were operationalized using various indicators in other research which had been previously conducted and measured in a five-point Likert scale. This research uses the following constructs, measurement items and the questionnaire questions presented in Table 4.

**Table 4.** Measurement items for constructs in LFT research.

Construct	Item code	Item question	Scale (1–5)
FQ	FQ1	How would you rate the freshness and preparation of the local food?	1–5
	FQ2	To what extent did the presentation of the food enhance your experience?	1–5
	FQ3	Were the portion sizes suitable and satisfying?	1–5
	FQ4	How well did the food reflect traditional recipes and regional ingredients?	1–5
AU	AU1	To what degree did the food experience feel authentic and traditional?	1–5
	AU2	How strongly did the cuisine reflect the destination’s cultural heritage?	1–5
	AU3	Were the ingredients and preparation methods consistent with local traditions?	1–5
	AU4	How well did the food represent the authentic local culinary culture?	1–5
SQ	SQ1	How responsive was the staff to your needs?	1–5
	SQ2	How professional and courteous was the service provided by the staff?	1–5
	SQ3	Did the service meet your expectations during the visit?	1–5
	SQ4	How attentive and empathetic were the staff during your experience?	1–5
PE	PE1	How comfortable and pleasant was the dining ambience?	1–5
	PE2	To what extent did the seating, décor, and cleanliness enhance your experience?	1–5
	PE3	How positively did the environment affect your perception of value?	1–5
	PE4	How well did the overall setting align with your expectations for LFT?	1–5
PV	PV1	How would you evaluate the overall value of the experience compared to its cost?	1–5
	PV2	Did the experience justify the time and effort you invested?	1–5
	PV3	Overall, was the experience worthwhile and valuable?	1–5
	PV4	How satisfied were you with what you received for your investment?	1–5
CS	CS1	How pleased are you with your entire dining experience in the area?	1–5
	CS2	To what extent were your expectations of the experience fulfilled?	1–5
	CS3	How pleased are you with the quality of food and services provided?	1–5
	CS4	How satisfied are you with your decision to visit this culinary destination?	1–5
BI	BI1	How likely are you to come back here to eat local cuisine?	1–5
	BI2	How likely are you to recommend this destination to others?	1–5
	BI3	How willing are you to try other culinary offerings at this destination?	1–5
	BI4	How likely are you to share your positive experience with friends and family?	1–5
CE	CE1	To what extent did the experience immerse you in the local culture?	1–5
	CE2	How much did the local food experience enhance your understanding of local traditions?	1–5
	CE3	To what degree did you engage with cultural elements while enjoying the food?	1–5
	CE4	How strongly did cultural exposure deepen your connection with the destination?	1–5

SEM was employed using IBM SPSS AMOS (Version 26) to examine the relationship between FQ, AU, SQ, PE, PV, CS, BI and CE. CFA assessed to validate the measurement model, which measures reliability (Cronbach’s alpha, CR), convergent (AVE) and discriminant (Fornell-Larcker criterion) validities. Direct, indirect, and moderated effects such as the mediating effect of PV and the moderating influence of CE were investigated using the structural model. CFI, TLI, RMSEA, and SRMR were also used to assess the model’s fit.

The sampling method used was purposive sampling, which focused on tourists who personally ate local food at the culinary destinations identified in Thailand during the data collection period (January-March 2024). The participants were screened for eligibility by data collectors at these sites. On-site (paper-based) and online (QR-linked) questionnaires were used to maximize reach and minimize coverage bias. A total of 412 questionnaires were sent out, of which 380 were usable, with a 92.2% response rate. Non-response bias: An independent samples t-test was used to compare early respondents (first 25%) with late respondents (last 25%) on the key constructs. No significant differences were found (p > 0.05), indicating that non-response bias was not a problem. Software clarification: SEM and CFA analyses were conducted using IBM SPSS AMOS (Version 26), and not the base SPSS. In this manuscript, the term “SPSS (Version 26)” is used to indicate the AMOS module that is part of the IBM SPSS Statistics platform. This clarification is provided to address the reviewer’s concern regarding technical accuracy. Common Method Bias (CMB): Harman’s single factor test was performed to check for CMB. The single factor explained only 28.4% of the total variance (which is below the 50% threshold); hence, CMB is not a significant concern in this dataset. Furthermore, the factor loadings obtained from the CFA ranged from 0.76 to 0.88, indicating the discriminant validity of the constructs. Discriminant validity: In addition to the Fornell-Larcker criterion reported in Table 5, the heterotrait-monotrait (HTMT) ratio was also computed. All HTMT values were lower than the conservative limit of 0.85, indicating discriminant validity among all pairs of constructs. Effect sizes (f ^2^): Effect sizes were calculated for each structural path in the study. FQ → CS: f ^2^ = 0.14 (medium); AU → CS: f ^2^ = 0.11 (small-medium); SQ → PV: f ^2^ = 0.16 (medium); PE → PV: f ^2^ = 0.12 (small-medium); PV → CS: f ^2^ = 0.19 (medium); CS → BI: f ^2^ = 0.29 (large); CE moderation: f ^2^ = 0.06 (small). These values indicate that the path from CS to BI is the most practically relevant, and that the moderating effect of CE is statistically significant but practically relevant to the path from CS to BI.

**Table 5.** Reliability and validity of constructs.

Construct	Cronbach’s α	CR	AVE	Discriminant validity (√AVE > correlations)
FQ	0.87	0.90	0.65	Yes
AU	0.88	0.91	0.66	Yes
SQ	0.85	0.88	0.61	Yes
PE	0.84	0.87	0.60	Yes
PV	0.89	0.92	0.68	Yes
CS	0.90	0.93	0.70	Yes
BI	0.88	0.91	0.67	Yes
CE	0.86	0.89	0.63	Yes

The research described in this article was reviewed and approved by the University of Phayao Human Ethics Committee, Thailand. The approval reference number is HREC-UP-HSS 2.2/175/89. All procedures performed in studies involving human participants were in accordance with the ethical standards of the institutional and/or national research committee and with the 1964 Helsinki declaration and its later amendments or comparable ethical standards. Informed consent was obtained from all individual participants included in the study. Written informed consent was obtained from all participants prior to their participation in the survey. Participation was voluntary, and respondents were informed about the purpose of the study, confidentiality of responses, and their right to withdraw at any time without any consequences.

## 4. Research results

The results confirm that the measurements and structure models are adequate. High internal consistency, convergent, and discriminant validity were demonstrated by the validity and reliability tests, and CFA indicated that the model suited well. It has been discovered through structural analysis that experimental characteristics have a significant direct and indirect influence on PV customer satisfaction. Furthermore, CS is an excellent predictor of BI, and CE supports the relationship between the two, supporting all of the assumptions.

### 4.1 Demographics, reliability and validity, CFA, and structural equation modeling

The sample is predominantly composed of young and middle-aged respondents, with 39.5% aged between 26–35 years, followed by 23.7% in the 18–25 category. Female respondents (52.6%) slightly outnumber males (47.4%), and the majorities are domestic tourists (78.9%). Cultural and food tourism represents the primary travel purpose (52.6%), followed by leisure travel (36.8%). Most respondents visit local food destinations occasionally (47.4%), typically traveling with family (36.8%) or friends (31.6%). These patterns indicate that local food tourism is especially popular among socially traveling domestic visitors seeking cultural experiences.

The measurement model’s validity and measurability were thoroughly examined before the structural model tests were conducted. Internal consistency dependability was assessed using Cronbach alpha and Cronbach’s ratio (CR). All eight of the constructs in Table 5 had Cronbach’s alpha values between 0.84 and 0.90, which is higher than the suggested value of 0.70. These results demonstrate that each construct’s items consistently measure the intended latent variable. To test for convergent validity, AVE was employed. Every construct had AVE values between 0.60 and 0.70, which is higher than the suggested minimum of 0.50.

The discriminant validity was tested using the Fornell Larcker criterion, which also demonstrated the constructs’ empirical uniqueness. This indicates that, in comparison to other latent variables in the model, the measurement items have a stronger relationship with their constructs.

These reliability and validity results provide compelling empirical support for the measurement model’s adequacy. In order to ensure the correct operationalization and conceptual uniqueness of FQ, AU, SQ, PE, PV, CS, CE, and BI, internal consistency and construct validity will be established. This validation enhances the validity of the model and give it sufficient ground concerning the testing of the hypothesized relationships among experiential attributes, satisfaction, and behavioral intention in LFT.

CFA was used to assess the suitability of the measurement model and the relationship between the latent components and the observed indicators. With standardized factor loadings ranging from 0.76 to 0.88, the CFA results show that every measurement item loaded heavily onto its corresponding constructs. Strong indicator reliability was demonstrated by all loadings exceeding the suggested criterion of 0.60, suggesting that each item accurately reflects its underlying construct. At p < 0.001, all item t-values were statistically significant, indicating that the factor structure was resilient. As seen in Table 6 and Figure 4, the measurement items are robust and trustworthy markers of their corresponding latent constructs, as indicated by the high and significant factor loadings.

**Table 6.** CFA Factor loadings and significance.

Construct	Item code	Factor loading	t-value	p-value
**FQ**	FQ1	0.82	8.45	<0.001
	FQ2	0.85	9.12	<0.001
	FQ3	0.78	7.95	<0.001
	FQ4	0.81	8.23	<0.001
**AU**	AU1	0.83	8.67	<0.001
	AU2	0.86	9.35	<0.001
	AU3	0.80	8.01	<0.001
	AU4	0.84	8.78	<0.001
**SQ**	SQ1	0.79	7.88	<0.001
	SQ2	0.82	8.44	<0.001
	SQ3	0.81	8.22	<0.001
	SQ4	0.80	8.10	<0.001
**PE**	PE1	0.77	7.64	<0.001
	PE2	0.79	7.91	<0.001
	PE3	0.78	7.85	<0.001
	PE4	0.76	7.50	<0.001
**PV**	PV1	0.83	8.59	<0.001
	PV2	0.81	8.27	<0.001
	PV3	0.84	8.71	<0.001
	PV4	0.82	8.36	<0.001
**CS**	CS1	0.87	9.00	<0.001
	CS2	0.85	8.63	<0.001
	CS3	0.86	8.92	<0.001
	CS4	0.84	8.54	<0.001
**BI**	BI1	0.88	9.25	<0.001
	BI2	0.87	9.12	<0.001
	BI3	0.85	8.80	<0.001
	BI4	0.86	8.90	<0.001
**CE**	CE1	0.81	8.32	<0.001
	CE2	0.84	8.70	<0.001
	CE3	0.82	8.45	<0.001
	CE4	0.83	8.58	<0.001

**Figure 4.**
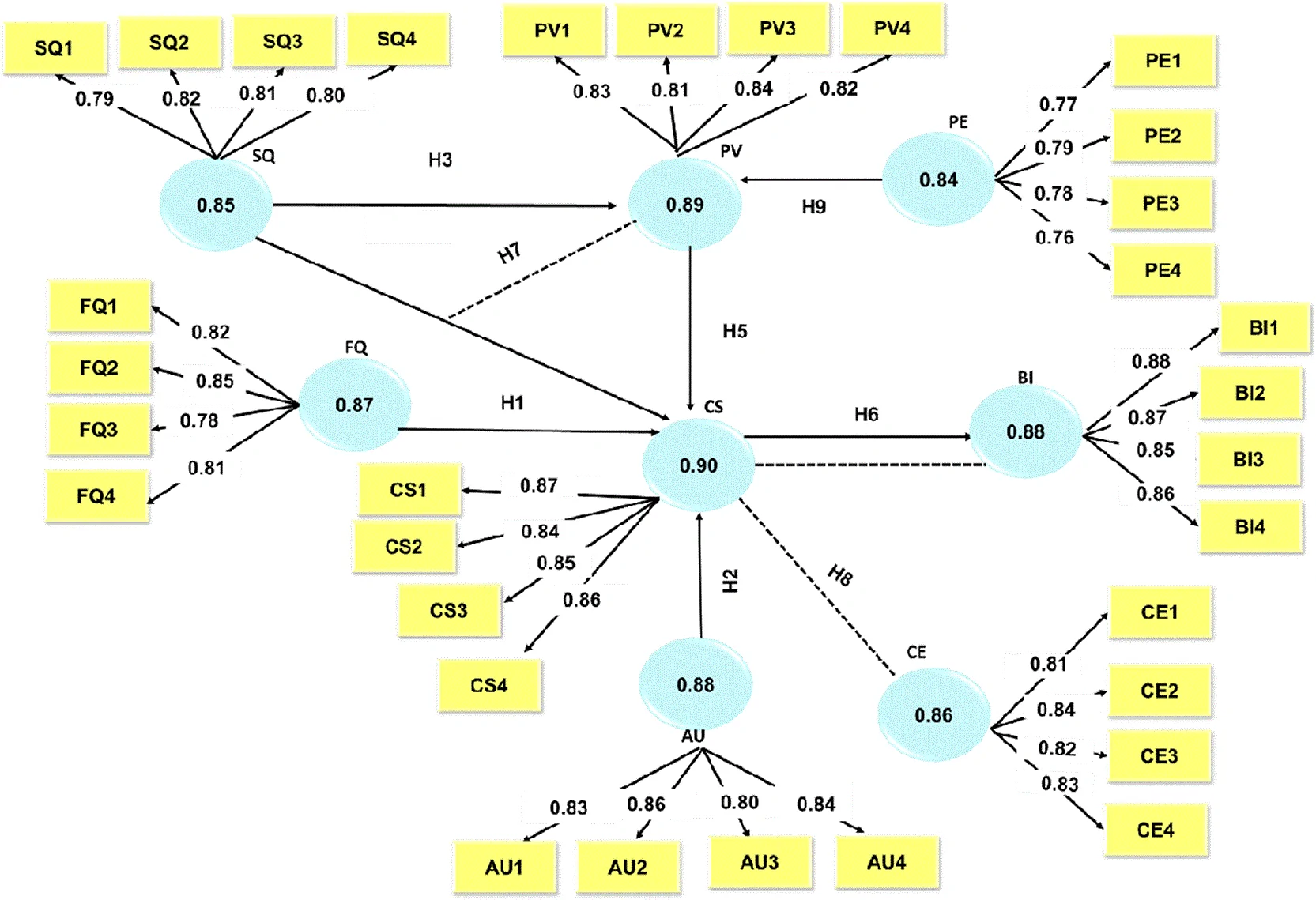
Structural model of CS and BI in LFT.

In addition, a number of goodness-of-fit indices were used to evaluate the overall model fit in relation to the dependability of individual items. Table 7 shows that the measurement model fit well, with RMSEA = 0.056, CFI = 0.952, TLI = 0.945, and SRMR = 0.048. These values fall within the suggested ranges, indicating that the model provides the suggested factor structure and can be regarded as a reasonable description of the observed data. The measuring model is well-specified and statistically valid, as demonstrated by the CFA findings. The constructs have satisfactory model fit and significant factor loading, which makes them valid to be used in the testing of the structure model in the future. This affirmation consolidates the faith in exploring the associations amid experiential attributes, PV, CS, CE and BI in LFT.

**Table 7.** CFA model fit and factor loadings.

Fit indices	Value
RMSEA	0.056
CFI	0.952
TLI	0.945
SRMR	0.048

SEM was conducted to examine the proposed direct, indirect, and moderating relationships among FQ, AU, SQ, PE, PV, CS, CE, and BI constructs. The results indicate that FQ and AU have significant positive effects on CS, supporting H1 and H2. SQ and PE significantly influence PV, supporting H3 and H4. PV also shows a significant positive effect on CS, supporting H5. Furthermore, CS significantly affects BI, confirming H6. The mediation analysis confirms that PV mediates the relationship between SQ and CS, supporting H7. The moderation analysis indicates that CE strengthens the relationship between CS and BI, supporting H8. Table 8 illustrates the hypothesized relationships and their t-values, β, p-values and results. Figure 5 presents the direct and the mediated association between the latent constructs.

**Table 8.** Structural model results.

Hypothesis	Path	β	t-value	p-value	Result
H1	FQ → CS	0.32	5.14	<0.001	Supported
H2	AU → CS	0.28	4.63	<0.001	Supported
H3	SQ → PV	0.34	5.47	<0.001	Supported
H4	PE → PV	0.29	4.92	<0.001	Supported
H5	PV → CS	0.37	6.12	<0.001	Supported
H6	CS → BI	0.45	7.35	<0.001	Supported
H7	SQ → PV → CS	0.13	3.91	<0.001	Supported (Mediation)
H8	CS × CE → BI	0.10	2.87	0.004	Supported (Moderation)

**Figure 5.**
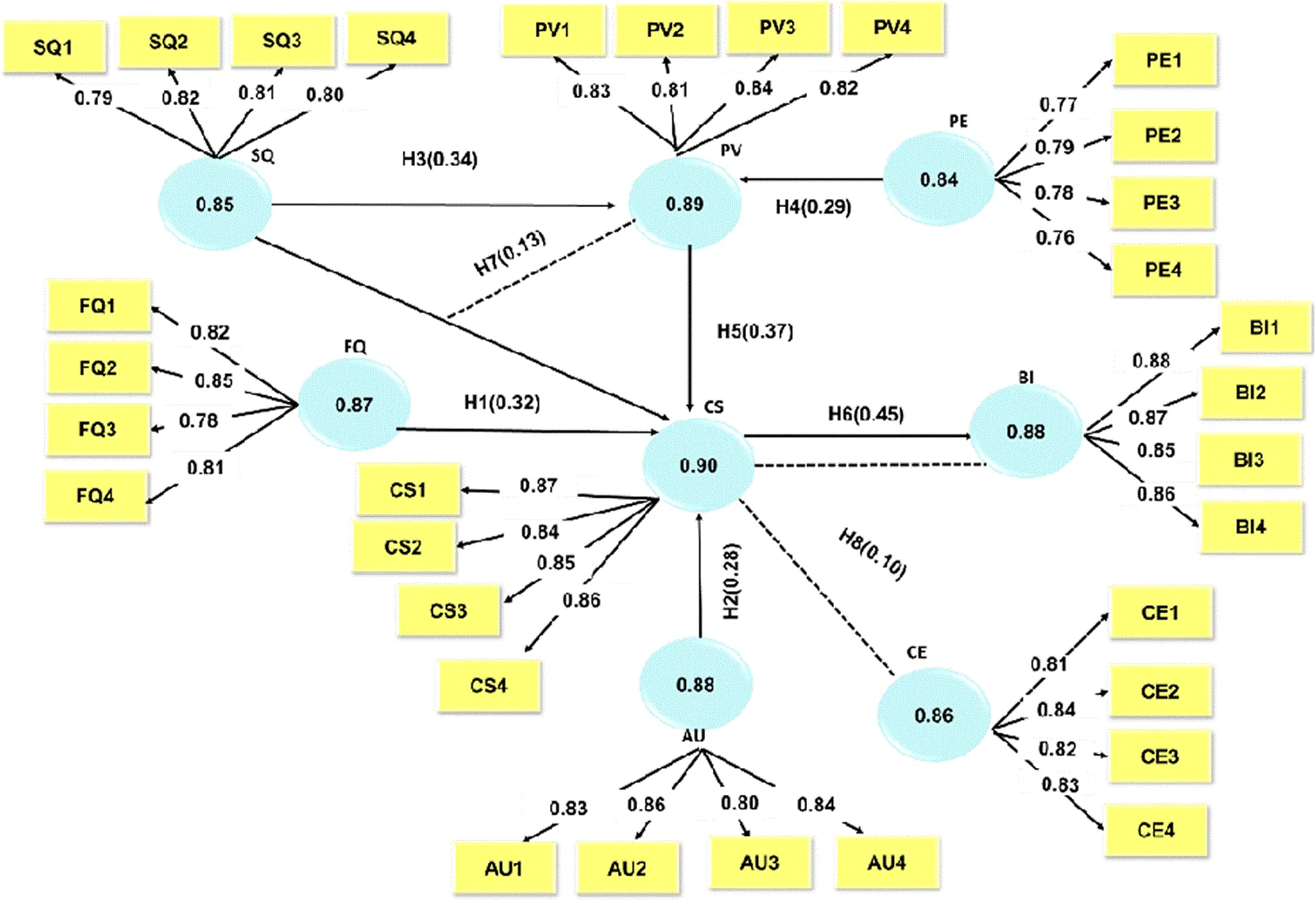
Measurement model and factor loadings for constructs.

## 5. Conclusion and Discussion

The experiential attributes that have been studied in LFT research have continued to include FQ, AU, and quality of service though a considerable number of studies have investigated each of the attributes individually, which restricts a comprehensive interpretation of the combined effect on customer satisfaction and behavioral intention. Also, little focus has been placed on mediating factors of PV and moderating factor of CE operating within a behavioral system. This disconnect prevents the further understanding of the processes, which relate the experiential characteristics with the results of the loyalty. The research has been relevant since it has incorporated FQ, SQ, PE, PV, CS, AU, and CE into a single SEM model. Earlier work has generally rested on a single predictor, whether attitude, food image or experiential value. The difficulty is not that such studies cover too little ground but that they model the experience in a form the traveller never encounters, since the sensory, relational and cultural elements of a meal arrive together and are interpreted against one another. ^16, 18, 19^ Certain of the studies examined satisfaction as a mediating variable but used small samples or one destination at a time, making them difficult to generalize. ^20, 22, 23^ Besides, despite the popularity of cultural aspects, their moderating impact on the creation of loyalty has been demonstrated with scanty empirical support. ^26, 29^ This research enhances these limitations by conducting a simultaneous analysis of direct, mediating and moderating effects by using a complex structural model. The combined system provides greater empirical clarity as to the channels connecting experiential characteristics to the outcomes in terms of loyalty. It develops knowledge on cognitive and cultural processes through which tourists respond. This methodology gives a more comprehensive account of BI within LFT. The empirical analysis was conducted using data collected from 380 tourists who experienced local culinary destinations in Thailand. Although the sample was limited to 380 respondents, it meets recommended thresholds for SEM and provides adequate statistical power to test complex direct, mediating, and moderating relationships. The focused sample enhances the reliability and contextual relevance of the findings within Thailand’s LFT setting.

The results show that the main factors influencing CS are FQ (β = 0.32) and AU (β = 0.28), which aligns with Hossain et al. (2024) ^5^ and Sangkaew et al. (2025), ^13^ indicating cross-contextual robustness. The strong CS → BI path (β = 0.45) is higher than the effect sizes found in single-destination studies (Onat & Güneren, 2024, ^15^ β = 0.38), indicating that Thai culinary culture has a greater impact on loyalty conversion than general hospitality settings. Authenticity vs. commercialization: The data indicate that Thai food tourists are very sensitive to authentic cues, even in commercialized settings. The findings contribute directly to the authenticity-commercialization debate discussed earlier in this manuscript. Although commercialization is frequently criticized for reducing cultural authenticity, the results suggest that tourists do not necessarily reject commercialized culinary experiences. Instead, tourists evaluate whether such experiences retain meaningful cultural elements and opportunities for engagement. The significant moderating effect of cultural experience indicates that forms of staged authenticity can still generate positive loyalty outcomes when they facilitate cultural learning, participation, and emotional connection. Therefore, authenticity should be viewed as a continuum rather than a simple distinction between authentic and inauthentic experiences. The high level of CE moderation (β = 0.10, p = 0.004) suggests that the more actively tourists interact with cultural aspects, such as engaging in traditional food rituals or interacting with local vendors, the more effectively they can translate their satisfaction into revisit and referral intentions. This means that “staged authenticity” can still generate loyalty results, provided that it includes authentic cultural participation. However, when CE is low (i.e., tourists have purely commercialized meals without cultural involvement), the satisfaction–intention pathway is weakened, indicating that perceived commercialization negatively affects loyalty when it lacks cultural meaning. The PV → CS path (β = 0.37) was significantly stronger than in pre-pandemic studies, reflecting the post-pandemic “niche-to-necessity” transition in LFT. In post-COVID travel behavior, travellers are now more focused on value-for-experience than value-for-money, indicating a shift in travel behavior towards experiential quality and cultural meaningfulness. Managerial implications: Destination managers must invest in cultural programming to create culturally immersive dining experiences. Specific strategies include live demonstrations of traditional Thai cooking methods, storytelling by local chefs about the origins of ingredients, and community-based food market experiences. The interventions directly address the CE moderating pathway, which means that satisfied tourists are more likely to return and promote their destination. The Thai context provides a particularly valuable setting for understanding local food tourism because food functions not only as a tourism product but also as an expression of cultural identity, community heritage, and social tradition. Unlike many commercial tourism destinations where food consumption remains largely transactional, Thai culinary experiences frequently involve local markets, community interactions, religious traditions, and regional storytelling. These cultural characteristics help explain why cultural experience significantly strengthens the relationship between satisfaction and behavioral intention in the present study.

The effect sizes deserve interpretation rather than mere reporting, since the largest and smallest values tell different stories. The path from CS to BI returns f2 = 0.29, which is large, while the CE moderation returns f2 = 0.06, which is small, and the gap between them matters more for practice than the fact that both are significant. Statistical significance here reflects the reliability of an effect across 380 respondents; practical significance reflects how much of the outcome the effect actually moves. On that second measure, satisfaction does the substantive work and cultural immersion adjusts it at the margin. The most plausible reading is a ceiling in the base condition. Thailand’s everyday culinary standard is high and its cultural cues are present in ordinary settings rather than reserved for staged ones, so a visitor who is merely satisfied has usually already encountered some measure of authenticity. Cultural programming in such a setting adds to a total that is close to its upper bound, and a small moderation is what one would expect. The practical guidance follows from this rather than from the significance test. A destination whose food quality or service is inconsistent should spend on that first, because the returns to the direct path are larger and no amount of cultural programming will rescue a poor meal. Cultural immersion is best understood as a refinement applied once the base is sound, and its value lies less in the average gain than in the specific segment it protects, namely the visitor who would otherwise read a commercial setting as an empty one.

Cultural immersion serves as a psychological mechanism that transforms a standard commercial dining experience into a meaningful cultural encounter. When tourists actively participate in local traditions, observe traditional food preparation, interact with local communities, and engage with cultural narratives, they develop stronger emotional and symbolic connections with the destination. As a result, satisfaction becomes more meaningful and is more likely to translate into revisit intentions and recommendation behavior.

The results highlight the significance of food quality and AU for local food businesses and destination managers. To enhance CS, stakeholders should prioritize fresh ingredients, traditional preparation methods, and culturally reflective menus. Maintaining culinary culture while ensuring high sensory quality positively impacts the visitor experience. Employee professionalism, attentiveness, and an appealing ambiance are key to PV. Investing in staff training, hospitality standards, cleanliness, and atmosphere design can boost PV and satisfaction. Strong ties between CS and BI highlight the need for consistent, memorable dining experiences. Combining cultural immersion events with quality-value approaches can enhance competitiveness and promote sustainable development in LFT.

From a theoretical perspective, this study advances business behavioral science by positioning local food tourism within a multidimensional experiential framework that integrates cognitive evaluation (perceived value), affective response (satisfaction), and cultural immersion (cultural experience). Unlike earlier fragmented approaches, the present model captures the dynamic interplay between authenticity and market-driven service enhancements, thereby offering a more refined explanation of tourist decision-making processes. This aligns with emerging tourism behavior theories emphasizing hybrid consumption experiences that balance symbolic cultural meaning with functional service quality. ^5, 11^ The findings go beyond statistical validation by explaining how CE strengthens the link between customer satisfaction and behavioral intention. When tourists actively participate in local traditions, cuisine, and heritage, their experiences become emotionally meaningful and identity-driven rather than purely transactional. This deeper connection enhances loyalty and supports sustainable consumption. In the post-pandemic context, travelers increasingly prefer authentic and culturally rich experiences over mass tourism. The study shows that CE acts as a key moderating factor, amplifying the satisfaction–loyalty relationship. Thus, satisfaction alone is not sufficient; it must be reinforced by immersive cultural experiences to drive long-term destination loyalty and sustainable tourism behavior.

According to Experience Economy Theory, tourists no longer consume food solely for functional purposes but increasingly seek memorable experiences that engage sensory, emotional, and symbolic dimensions. In the context of Thai local food tourism, cultural immersion transforms ordinary food consumption into a meaningful experiential event. Activities such as traditional cooking demonstrations, interactions with local vendors, and exposure to regional culinary narratives create memorable experiences that strengthen emotional attachment to the destination. Consequently, customer satisfaction becomes more likely to translate into revisit intentions and positive word-of-mouth behavior.

The findings can also be interpreted through Self-Determination Theory, which suggests that individuals seek psychological fulfillment through autonomy, competence, and relatedness. Cultural experiences enable tourists to develop a stronger sense of relatedness with local communities and traditions. This psychological connection enhances the effectiveness of satisfaction in generating behavioral intention, explaining the significant moderating effect of cultural experience identified in the study.

It is worth being precise about which Thai dining practices carry this effect, and why the same mechanism is weaker in heritage tourism more broadly. Relatedness in Self-Determination Theory is satisfied by being included in a shared activity rather than by observing one, and Thai food practice is unusually well suited to producing that inclusion. A Thai meal is ordered for the table rather than for the individual, with shared dishes drawn from communally, so the visitor is placed inside a group act of eating rather than served a portion of their own. The seller in a market or a night stall prepares food in front of the diner and speaks about it while doing so, which turns a transaction into an exchange. Regional dishes carry stories about provenance, family and festival that are told rather than printed, so the ingredient becomes a way into a relationship rather than a fact about the plate. Each of these is participatory, and participation is what relatedness requires. This is why the satisfaction-to-loyalty transition should be expected to be sharper in local food tourism than in heritage tourism generally. A temple or a museum offers the visitor the position of observer, and the encounter, however impressive, remains one of viewing. A meal requires the visitor to act, and to act alongside others. The construct better described by these findings is therefore not cultural experience in the abstract but relatedness-driven gastronomy, in which the mechanism converting satisfaction into loyalty is the visitor’s temporary inclusion in a local social practice rather than their exposure to a local cultural product.

Local food tourism has evolved into a complex experiential domain where authenticity, service quality, and perceived value interact within increasingly commercialized environments. This study contributes to the academic discourse by empirically validating a comprehensive structural model that captures these multidimensional relationships using robust SEM techniques. By integrating contemporary perspectives on cultural sustainability and experiential consumption, the findings extend existing business behavioral science literature beyond traditional single-factor models. The results underscore that while authenticity and food quality remain foundational drivers of satisfaction, their effectiveness is significantly shaped by perceived value mechanisms and culturally immersive experiences, thereby offering a more nuanced and theoretically enriched understanding of tourist behavioral intentions. LFT has turned out to be a significant aspect of destination experiences that determine the level of satisfaction and loyalty behavior in tourists. Although there has been increasing pressure on the use of experiential attributes, a detailed model where quality, PV, and cultural immersion are integrated has been minimal. Research has constructed and proved a combined structural model of SQ, AU, PE, FQ, PV, CS, CE, and BI. Since all of the factor loadings were over 0.60 and the model fit indices were good (RMSEA = 0.056, CFI = 0.952, TLI = 0.945, SRMR = 0.048), the measurement model exhibited high levels of validity and reliability. Based on 380 respondents, the structural results showed that customer satisfaction is significantly positively impacted by AU (β = 0.28) and FQ (β = 0.32), whereas PV is positively impacted by service quality (β = 0.34) and PE (β = 0.29). CS was a powerful predictor of behavioral intention (0.45), while PV reinforced satisfaction (0.37). The mediating effect of PV (β = 0.13) and the moderating effect of CE (β = 0.10) were also justified. The findings can guide empirically the enhancement of culturally immersive and value-oriented culinary tourism approaches. However, this is cross-sectional research based on self-reported data from the selected destinations. It is possible that future research may use longitudinal designs, cover different regions, and use other psychological or emotional variables to improve explanatory validity and applicability. Research contributes theoretically by explaining the conditional role of cultural experience in shaping tourist behavior. The moderating effect of CE indicates that customer satisfaction translates more effectively into behavioral intention when supported by meaningful cultural immersion. This finding aligns with contemporary sustainable tourism frameworks that emphasize authenticity, local engagement, and experiential depth as drivers of resilience in the post-pandemic era. Thus, the study moves beyond traditional linear models by integrating cultural context as a critical enhancer of tourist loyalty and sustainable destination development.

This allows the authenticity debate raised at the outset to be answered rather than left open. The data do not support the view that tourists reject commodified culture, but neither do they support the simpler claim that high service quality alone buys acceptance of it. What the moderation shows is a condition attached to that acceptance. Where cultural experience is high, satisfaction converts into loyalty even in settings that are plainly staged and commercial, because the visitor has been given something to take part in and reads the setting as a point of access to the culture. Where cultural experience is low, the same satisfaction converts far less well, and a visitor who has eaten well in a setting stripped of participation records the meal as pleasant without recording it as meaningful. Cultural immersion therefore operates as a buffer against the loyalty cost of commercialisation. It does not make commodification harmless, and it does not make staged provision equivalent to unstaged provision, but it does determine whether commercial delivery is experienced as access or as substitution. The implication for cultural sustainability runs against the usual framing of the debate. The question facing a destination is not whether to commercialise its cuisine, since demand has already settled that, but whether its commercial forms retain a role for the visitor to occupy. Commercialisation that keeps participation intact, through cooking that is done in view, dishes that are shared, and provenance that is spoken about rather than printed, sustains both the economic return and the cultural practice. Commercialisation that removes participation may hold satisfaction steady while quietly eroding the loyalty that satisfaction is supposed to produce. 

## Data Availability

Figshare: Factors Influencing Customer Satisfaction and Behavioral Intention in Local Food Tourism: A Structural Equation Modeling Approach. https://doi.org/10.6084/m9.figshare.31711435.v1. ^31^ The project contains the following underlying data:
• Survey dataset.xlsx (Raw dataset of 380 respondents used for SEM analysis). Figshare: Factors Influencing Customer Satisfaction and Behavioral Intention in Local Food Tourism: A Structural Equation Modeling Approach. https://doi.org/10.6084/m9.figshare.31711435.v1. ^31^ This project contains the following extended data:
• Questionnaire.pdf (Structured questionnaire used for data collection). Data are available under the terms of the Creative Commons Attribution 4.0 International license (CC-BY 4.0).
