# Supplementary material for: Factors Influencing Customer Satisfaction and Behavioral Intention in Local Food Tourism: A Structural Equation Modeling Approach
Source: F1000Res. 2026 Aug 21;15:596. Originally published 2026 Apr 19. [Version 5] doi: 10.12688/f1000research.179249.5 (PMC13560856; doi:10.12688/f1000research.179249.5)
Supplement: Peer review history

# Reviewer report: Reviewer response for version 4

**DOI:** 10.5256/f1000research.204379.r506181

**Published:** 2026-08-12

**Authors:** Regina Jokom

**Affiliations:** 1 Petra Christian University, Surabaya, Indonesia

The topic is relevant and the analysis is carefully done. A few points would benefit from further attention:

**1. The type of tourist (Domestic or international Tourist?). **The Results indicate that 78.9% of respondents were domestic tourists, but this is not clearly analyzed. The distinction matters for most constructs in the model. For example, domestic visitors already share the culinary culture under study, so authenticity and cultural experience are likely to mean something different to them. While, international visitors found that local food is unfamiliar and novelty. This may also help explain the small moderating effect of cultural experience. Please interpret the findings explicitly from the perspective of a predominantly domestic sample. Otherwise, this should be acknowledged as a clear limitation, and the conclusions.

**2. Rationale for combining two theories. **The manuscript draws on both Experience Economy Theory and Self-Determination Theory, but the reason for combining them is not clearly explained. It is not clear whether Experience Economy Theory alone could explain the proposed relationships, or what Self-Determination Theory adds to the first theory. Please clarify whether the study confirms existing theory or extends it in some way.

**3. Framework and main concept. **The research model brings together many factors, but they are presented largely as a list of predictors rather than as an argument. The paper would be considerably stronger if these factors were framed within one concept that gives the study a clear point of emphasis. For example, a specific theoretical development, or the distinctive characteristics of the Thai context that make this model more meaningful. Please also consider whether authenticity alone is sufficient to answer the commercialization-versus-tradition debate raised in the Introduction, or whether an additional construct or perspective is needed.

**4. The measurement items.** Section 3.1 states that the constructs were measured "according to the validated scales", but the manuscript does not identify where the items came from. Please state the origin of each construct's items, and indicate what was modified where items were adapted.

**5. The Discussion concentrates almost entirely on authenticity and cultural aspects. **Physical environment, service quality and perceived value receive little attention despite being part of the model and having supported paths. The Discussion should explain what each construct contributes to the overall explanation, otherwise a more parsimonious model would be sufficient. Therefore, please expand the discussion of the remaining paths, particularly dining ambience, so that the interpretation corresponds to the model actually tested. In Addition, only a few sources are cited in this section. Please add further references, both those that support the arguments and those that contradict the present findings.

**6. Study site.** The sample is described only as "tourists in Thailand" (Section 3.1). Please name the city or the specific culinary destinations. Given that most respondents are domestic, identifying the site would be more sharper in contextual analysis.

**7. Figure 1.** Cultural Experience is shown with the same elements as Service Quality (friendly staff, responsiveness, hospitality), which conflicts with Table 3 and Table 4 and blurs the distinction between the two constructs. Please correct the figure.

**8. Table 2** (H8) also labels CE as "Customer Engagement" rather than "Cultural Experience". One term should be used throughout.

**9. Interpretation of the moderating effect.** The text refers to "the high level of CE moderation", yet the explanation in f² result indicate a small effect. Please align the wording with the magnitude of the estimate.

**10. Claims regarding sustainable behaviour.** The Discussion moves from satisfaction and behavioural intention to sustainable consumption and sustainable tourism behaviour, but there is no sustainability-related behaviour was measured. Please make the reasoning explicit or moderate these statements so they remain within what the data support.

Is the work clearly and accurately presented and does it cite the current literature?

Partly

If applicable, is the statistical analysis and its interpretation appropriate?

Yes

Are all the source data underlying the results available to ensure full reproducibility?

Yes

Is the study design appropriate and is the work technically sound?

Yes

Are the conclusions drawn adequately supported by the results?

Partly

Are sufficient details of methods and analysis provided to allow replication by others?

Yes

Reviewer Expertise:

Marketing and Tourism Management

I confirm that I have read this submission and believe that I have an appropriate level of expertise to confirm that it is of an acceptable scientific standard, however I have significant reservations, as outlined above.

# Reviewer report: Reviewer response for version 4

**DOI:** 10.5256/f1000research.204379.r507971

**Published:** 2026-08-11

**Authors:** Senthilkumaran Piramanayagam

**Affiliations:** 1 Manipal Academy of Higher Education, Udupi, Karnataka, India

Thank you for the opportunity to review this scholarly work. The manuscript is well-structured and demonstrates academic rigor. The identified methods are well-suited to the study. Justification for the choice of the research problem, a relevant review of the literature, a method section, statistical analysis, and a supporting argument for its novelty and contribution make this work acceptable for indexing.

 I have a few suggestions:

 1. As the statements in the scale in the form of questions (How would you rate the freshness and preparation of the local food?, To what extent did the presentation of the food enhance your experience?, and so on), authors need to describe how the five-point Likert scale has been modified to capture the response.

 2. The eligibility criteria for the selection of participants using purposive sampling need to be included in the methodology section.

 3. Statements like this need to be removed in the final manuscript. "This clarification is provided to address the reviewer’s concern regarding technical accuracy. Common Method Bias (CMB):" 

 4. Inclusion of the limitations of the current study and scope for further research.

Is the work clearly and accurately presented and does it cite the current literature?

Yes

If applicable, is the statistical analysis and its interpretation appropriate?

Yes

Are all the source data underlying the results available to ensure full reproducibility?

Yes

Is the study design appropriate and is the work technically sound?

Yes

Are the conclusions drawn adequately supported by the results?

Yes

Are sufficient details of methods and analysis provided to allow replication by others?

Partly

Reviewer Expertise:

Consumer Behaviour in Hospitality and Tourism Research

I confirm that I have read this submission and believe that I have an appropriate level of expertise to confirm that it is of an acceptable scientific standard.

# Reviewer report: Reviewer response for version 4

**DOI:** 10.5256/f1000research.204379.r496866

**Published:** 2026-07-01

**Authors:** Kittisak Wongmahesak

**Affiliations:** 1 Publication Research Institute and Community Service, Universitas Muhammadiyah Sidenreng Rappang (Ringgold ID: 605937), Sidenreng Rappang Regency, South Sulawesi, Indonesia; 2 Post-Doctoral Fellowship Research Collaboration Program, Lincoln University College (Ringgold ID: 283706), Petaling Jaya, Selangor, Malaysia; 3 Faculty of Political Science, North Bangkok University, Sai Mai, Bangkok, Thailand

The author has made significant strides in addressing the technical and theoretical concerns raised in previous rounds of review, particularly regarding the integration of Experience Economy Theory and Self-Determination Theory (SDT). The inclusion of effect sizes (f^2) and the clarification of the statistical software used (IBM SPSS AMOS) provide the transparency expected of a high-tier international publication such as F1000Research. However, from a critical academic standpoint, there remain subtle yet vital areas where the manuscript requires "final sharpening" to truly elevate its contribution from a technically correct report to a sophisticated piece of behavioral science literature.

 The theoretical framework, while now anchored in SDT and Experience Economy, still requires a more seamless synthesis between the empirical findings and the underlying psychological mechanisms. The author argues that cultural experience (CE) serves as a moderator, yet the narrative transition from "satisfaction" to "loyalty" via "relatedness" (per SDT) feels somewhat mechanistic. In the Discussion, the author should offer a sharper argument about how specific Thai food rituals—such as the communal nature of Thai dining or the storytelling surrounding ingredients—facilitate a psychological sense of relatedness. Currently, the explanation of cultural immersion as a catalyst is present. Still, it lacks the "theoretical edge" that explains why this transition is more pronounced in local food tourism than in general heritage tourism. Strengthening this bridge would clarify the "So What?" factor for a global audience, positioning the Thai context not just as a case study, but as a model for "Relatedness-Driven Gastronomy."

 Regarding the methodological rigor, the transition to reporting effect sizes (f^2) is a commendable addition. However, the interpretation of these sizes in the Discussion remains slightly superficial. For instance, the path from Customer Satisfaction (CS) to Behavioral Intention (BI) yields a large effect size (0.29), while the CE moderation is relatively small (0.06). A sophisticated quantitative critique would explain why, despite its statistical significance, the moderator's practical influence is smaller than that of the direct path. In a high-authenticity environment like Thailand, is the "base quality" of the food so high that cultural immersion provides only incremental gains? Addressing the "practical significance" versus "statistical significance" of these varying effect sizes would demonstrate a mastery of advanced quantitative interpretation and provide more nuanced guidance for destination managers.

 The "critical debate" surrounding authenticity versus commercialization introduced in the Introduction is a highlight of this version, yet it lacks a strong resolution in the concluding sections. The author correctly identifies authenticity as a continuum, but the paper would benefit from a more concrete argument regarding "staged authenticity." Based on the data, does the author suggest that as long as the service quality is high, tourists are willing to accept a "commodified" version of Thai culture? This is a fundamental question in contemporary tourist behavior. By explicitly linking the moderation of CE to this debate, the author could argue that cultural immersion serves as a "buffer," allowing tourists to perceive value even in commercialized settings. This would transform the conclusion from a restatement of results into a sharp, original contribution to the global discourse on cultural sustainability.

 Finally, while the linguistic quality has improved, the manuscript still suffers from occasional "report-like" phrasing that disrupts the academic flow. The transition between the Literature Review and the Hypothesis Development needs to be more fluid, shifting from a summary of others' work to an argumentative synthesis that makes the current study feel inevitable. For instance, instead of merely stating that "earlier research usually narrowed down to a single predictor," the author should argue why single-predictor models are conceptually insufficient to capture the holistic "foodscape" of a post-pandemic traveler. This shift in tone from descriptive to argumentative is what distinguishes top-tier scholarship. Ensuring that the acronym "BI" is strictly and consistently defined—and that sentence fragments in the Abstract are corrected—will be the final polish required for indexing. If these analytical and narrative refinements are integrated, the manuscript will stand as a robust and theoretically enriched contribution to the field of gastronomy tourism.

Is the work clearly and accurately presented and does it cite the current literature?

Partly

If applicable, is the statistical analysis and its interpretation appropriate?

Yes

Are all the source data underlying the results available to ensure full reproducibility?

Yes

Is the study design appropriate and is the work technically sound?

Yes

Are the conclusions drawn adequately supported by the results?

Yes

Are sufficient details of methods and analysis provided to allow replication by others?

Yes

Reviewer Expertise:

Political Science, Public & Private Management, International Political Economy, Social Research

I confirm that I have read this submission and believe that I have an appropriate level of expertise to confirm that it is of an acceptable scientific standard, however I have significant reservations, as outlined above.

# Reviewer report: Reviewer response for version 3

**DOI:** 10.5256/f1000research.202732.r491862

**Published:** 2026-06-12

**Authors:** Sohel Rana

**Affiliations:** 1 University of Scholars, Dhaka, Bangladesh

I appreciate the authors' efforts in revising the manuscript and responding comprehensively to the reviewers' comments. The authors have adequately addressed the concerns raised during the review process. The manuscript is now methodologically sound, theoretically grounded, and suitable for publication. I therefore recommend acceptance for indexing.

Is the work clearly and accurately presented and does it cite the current literature?

Partly

If applicable, is the statistical analysis and its interpretation appropriate?

Yes

Are all the source data underlying the results available to ensure full reproducibility?

Yes

Is the study design appropriate and is the work technically sound?

Yes

Are the conclusions drawn adequately supported by the results?

Yes

Are sufficient details of methods and analysis provided to allow replication by others?

Partly

Reviewer Expertise:

Tourism, Marketing, Consumer Behavior.

I confirm that I have read this submission and believe that I have an appropriate level of expertise to confirm that it is of an acceptable scientific standard.

# Reviewer report: Reviewer response for version 3

**DOI:** 10.5256/f1000research.202732.r491861

**Published:** 2026-06-10

**Authors:** Kittisak Wongmahesak

**Affiliations:** 1 Publication Research Institute and Community Service, Universitas Muhammadiyah Sidenreng Rappang (Ringgold ID: 605937), Sidenreng Rappang Regency, South Sulawesi, Indonesia; 2 Post-Doctoral Fellowship Research Collaboration Program, Lincoln University College (Ringgold ID: 283706), Petaling Jaya, Selangor, Malaysia; 3 Faculty of Political Science, North Bangkok University, Sai Mai, Bangkok, Thailand

The current iteration demonstrates a significantly higher level of technical transparency and methodological rigor. Specifically, the explicit mention of IBM SPSS AMOS for structural modeling, the reporting of effect sizes (f^2), and the inclusion of Common Method Bias (CMB) testing through Harman’s single-factor test provide the necessary scientific integrity for a high-impact platform like F1000Research. The study effectively bridges the gap between technical competence and psychological precursors in the context of Thai local food tourism, which is particularly relevant in the post-pandemic "niche-to-necessity" evolution of the industry.

 From a critical academic perspective, the manuscript still needs final sharpening to move beyond technical accuracy to a leading theoretical contribution. While the integration of Experience Economy Theory and Self-Determination Theory is a step forward, the connection between the empirical results and these frameworks remains somewhat descriptive. The author confirms that Cultural Experience (CE) acts as a moderator, but the discussion needs to be more analytical regarding the "why." In the context of Thailand, the author should explain how cultural immersion turns a standard commercial meal into a behavioral trigger for revisit and referral. Furthermore, the narrative cohesion between the "Critical Debate" on authenticity introduced in the Introduction and the final Discussion needs a stronger bridge to explain if tourists are willing to overlook "staged authenticity" when sensory quality is high.

 The manuscript still has persistent linguistic issues and grammatical inaccuracies that affect its academic professionalism. There are significant instances of sentence fragments, most notably in the Abstract, where a crucial statement regarding research gaps lacks a main verb, leaving the reader to infer the intended meaning. Additionally, the text is marred by frequent comma splices and repetitive phrasing, such as the redundant use of "demanding" at the opening of the Introduction, which disrupts the prose's rhythmic flow. A critical technical oversight is the inconsistent use of the acronym "BI," which the author uses interchangeably with "Behavioral Intention" and "Business Intelligence." These inconsistencies are unacceptable for indexing and indicate a need for careful proofreading. The manuscript requires a comprehensive linguistic overhaul by a professional academic editor to ensure that the "Academic Flow" and "Sophisticated Prose" meet the standards of top-tier international publishing.

 In conclusion, the paper is technically sound, and the data are rich. Final approval will depend on deepening the theoretical dialogue and achieving linguistic excellence. Please move beyond restating statistical tables and instead offer a sharp, cohesive argument that highlights the study's specific Thai nuances. Consolidating the visuals and resolving the acronym overlap will make the academic narrative clearer and ensure the work is accessible to the global tourism research community. Addressing these analytical and linguistic points is essential for the article to be considered scientifically robust and ready for final indexing.

Is the work clearly and accurately presented and does it cite the current literature?

Partly

If applicable, is the statistical analysis and its interpretation appropriate?

Yes

Are all the source data underlying the results available to ensure full reproducibility?

Yes

Is the study design appropriate and is the work technically sound?

Yes

Are the conclusions drawn adequately supported by the results?

Yes

Are sufficient details of methods and analysis provided to allow replication by others?

Yes

Reviewer Expertise:

Political Science, Public & Private Management, International Political Economy, Social Research

I confirm that I have read this submission and believe that I have an appropriate level of expertise to confirm that it is of an acceptable scientific standard, however I have significant reservations, as outlined above.

# Reviewer report: Reviewer response for version 2

**DOI:** 10.5256/f1000research.200745.r482388

**Published:** 2026-05-22

**Authors:** Sohel Rana

**Affiliations:** 1 University of Scholars, Dhaka, Bangladesh

1. The manuscript lacks a strong theoretical underpinning. Although hypotheses are presented, the study does not sufficiently anchor the model within an established theory. Currently, the framework appears largely descriptive and variable-driven rather than theory-driven.

 2. The literature review mainly summarizes prior studies but lacks critical synthesis. Explain how this study differs from earlier SEM-based tourism studies. And develop a stronger research gap statement.

 3. Several methodological details are missing or underdeveloped: Sampling Procedure, CMB, Non-response Bias, and discriminant validity.

 4. Strengthen the discussion by interpreting findings theoretically, comparing findings with previous studies, and explaining managerial significance more analytically.

 5. The manuscript contains numerous grammatical and stylistic problems. Improve academic writing and language quality.

Is the work clearly and accurately presented and does it cite the current literature?

Partly

If applicable, is the statistical analysis and its interpretation appropriate?

Yes

Are all the source data underlying the results available to ensure full reproducibility?

Yes

Is the study design appropriate and is the work technically sound?

Yes

Are the conclusions drawn adequately supported by the results?

Yes

Are sufficient details of methods and analysis provided to allow replication by others?

Partly

Reviewer Expertise:

Tourism, Marketing, Consumer Behavior.

I confirm that I have read this submission and believe that I have an appropriate level of expertise to confirm that it is of an acceptable scientific standard, however I have significant reservations, as outlined above.

# Reviewer report: Reviewer response for version 2

**DOI:** 10.5256/f1000research.200745.r486400

**Published:** 2026-05-21

**Authors:** Kittisak Wongmahesak

**Affiliations:** 1 Publication Research Institute and Community Service, Universitas Muhammadiyah Sidenreng Rappang (Ringgold ID: 605937), Sidenreng Rappang Regency, South Sulawesi, Indonesia; 2 Post-Doctoral Fellowship Research Collaboration Program, Lincoln University College (Ringgold ID: 283706), Petaling Jaya, Selangor, Malaysia; 3 Faculty of Political Science, North Bangkok University, Sai Mai, Bangkok, Thailand

The revised manuscript (Version 2) demonstrates a commendable effort by the author to address initial concerns about the integration of contemporary literature and the narrative's structural flow. The inclusion of recent scholarship from 2025 (e.g., Phanpanya et al.; Walailak et al.; Worrachananun) significantly bolsters the study’s relevance, positioning it within the post-pandemic paradigm of "niche-to-necessity" culinary tourism. However, from the perspective of an advanced quantitative researcher and a professor of tourism, I find that while the technical execution is robust, the manuscript requires further "sharpening" to achieve the level of novelty and theoretical contribution expected by a top-tier international outlet like *F1000Research*.

**1. Theoretical Refinement and Novelty (The "So What?" Factor):** The primary challenge of this paper remains its incremental novelty. The paths between Food Quality (FQ), Service Quality (SQ), and Customer Satisfaction (CS) are well-trodden in tourism literature. To strengthen the contribution, the author should more strongly highlight the Moderating Role of cultural experience (CE). While H8 is supported, the Discussion section still treats CE as just another variable. The author should elaborate on *why* cultural immersion serves as a catalyst, transforming a standard meal into a behavioral trigger (revisit/referral). The "novelty" lies not in the fact that these variables interact, but in the specific cultural nuances of the Thai LFT context that make this interaction unique compared to Western or purely commercial dining contexts.

**2. Advanced Quantitative Rigor and Methodological Transparency:** While the SEM indices (CFI, TLI, RMSEA) are excellent, two technical omissions need to be immediately flagged:

 - Software Clarification: The author mentions using "SPSS (Version 26)" for SEM and CFA. Strictly speaking, base SPSS does not perform SEM; this is typically done via IBM SPSS AMOS, LISREL, or R (lavaan). The author should clarify whether AMOS was used, as this is a matter of technical accuracy.

 - Common Method Bias (CMB): Given that the data is cross-sectional and self-reported from a single source, the risk of CMB is high. The author must report the results of Harman’s Single Factor Test or, ideally, a Common Method Latent Factor test. Without addressing CMB, the validity of the structural paths—particularly the high correlation between satisfaction and behavioral intention—remains scientifically vulnerable.

 - Effect Size (f^2): While p-values indicate significance, the author should report effect sizes (f^2) to demonstrate the practical significance of each path. This would provide a more nuanced interpretation of which "experiential quality" is the most potent driver of loyalty.

**3. Narrative Cohesion and the "Essay" Style:** The manuscript has improved, but still retains a "report-like" structure in the Results and Methodology sections. The transition between the Literature Review and the Hypotheses Development should be more fluid. Specifically, the "Critical Debate" regarding authenticity versus commercialization introduced in the Introduction should be revisited in the Discussion. Does the data suggest that tourists in Thailand are willing to overlook "staged authenticity" if the service quality is high? Or does the moderation of CE imply that once authenticity is lost, satisfaction no longer leads to behavioral intention? Answering these questions in cohesive prose will satisfy the previously requested "Academic Flow" requirement.

**4. Strategic Recommendations for Final Polish:**

 - Deepen the Discussion: Move beyond restating Table 8. Contrast your findings with the "niche-to-necessity" evolution. If LFT is now a necessity, how does the role of "Perceived Value" (H5) shift compared to pre-pandemic studies?

 - Consolidate Visuals: Table 4 (Measurement Items) is quite long. Consider moving it to an Appendix or a supplementary file to allow the "Literature Review" and "Methods" sections to breathe as a continuous academic narrative.

 - Refine the Abstract: The abstract mentions "Business Intelligence (BI)" as a variable, but the paper uses "Behavioral Intention (BI)." Please correct the acronym overlap to avoid confusion for indexing services.

 In conclusion, the manuscript is technically sound, and the data are rich. However, to transition from a "technically correct" paper to indexing, the author must emphasize the theoretical contribution of the moderation effects and ensure methodological transparency regarding CMB and software usage. Addressing these points will sharpen the manuscript’s contribution to the global discourse on sustainable and cultural gastronomy.

Is the work clearly and accurately presented and does it cite the current literature?

Partly

If applicable, is the statistical analysis and its interpretation appropriate?

Yes

Are all the source data underlying the results available to ensure full reproducibility?

Yes

Is the study design appropriate and is the work technically sound?

Yes

Are the conclusions drawn adequately supported by the results?

Yes

Are sufficient details of methods and analysis provided to allow replication by others?

Yes

Reviewer Expertise:

Political Science, Public & Private Management, International Political Economy, Social Research

I confirm that I have read this submission and believe that I have an appropriate level of expertise to confirm that it is of an acceptable scientific standard, however I have significant reservations, as outlined above.

# Reviewer report: Reviewer response for version 1

**DOI:** 10.5256/f1000research.197745.r476970

**Published:** 2026-04-23

**Authors:** Kittisak Wongmahesak

**Affiliations:** 1 Publication Research Institute and Community Service, Universitas Muhammadiyah Sidenreng Rappang (Ringgold ID: 605937), Sidenreng Rappang Regency, South Sulawesi, Indonesia; 2 Post-Doctoral Fellowship Research Collaboration Program, Lincoln University College (Ringgold ID: 283706), Petaling Jaya, Selangor, Malaysia; 3 Faculty of Political Science, North Bangkok University, Sai Mai, Bangkok, Thailand

This article explores the determinants of customer satisfaction (CS) and behavioral intention (BI) within the local food tourism (LFT) sector in Thailand using Structural Equation Modeling (SEM). While the technical execution of the research is sound and the data analysis is rigorous, the manuscript requires significant refinement in its narrative structure and integration of the literature to meet the high academic standards of F1000Research.

**1) Narrative Structure and Presentation:** The current manuscript is heavily segmented with numerous subheadings and a high volume of figures and tables. To improve readability and academic flow, the authors should transition toward a more cohesive "prose" style. Specifically, the content should be streamlined into five core sections: Introduction, Literature Review, Research Methods, Research Results, and Conclusion and Discussion. Paragraphs should be balanced to ensure a smooth transition of arguments rather than relying on bullet points or excessive subdivisions.

**2) Literature Review and Argumentation:** The theoretical foundation needs more "sharpness" regarding the contemporary evolution of culinary tourism. The authors must demonstrate a deeper understanding of post-pandemic shifts and service quality dynamics. To achieve high-quality literature synthesis, the following recent works must be integrated into the discussion:

 - The discussion on service quality and the marketing mix should be bolstered by citing Walailak et al. (2025), specifically regarding how service quality factors drive satisfaction in the Thai hospitality context.

 - To address the resilience of the tourism sector and the shift from "niche" to "necessity," the authors must incorporate the multidimensional evolution of culinary tourism as discussed by Phanpanya et al. (2025).

 - The argument regarding the impact and opportunities of food tourism in the modern era should be strengthened using the insights on sustainability provided by Worrachananun (2025).

**3) Integration of Results and Discussion:** While the SEM results are clearly presented in Table 8 and Figure 8, the "Conclusion and Discussion" section should do more than restate the statistical findings. It must explain why specific variables, such as Cultural Experience (CE), serve as moderators and how these findings contrast with or support the newly suggested literature on sustainable food tourism and post-pandemic resilience.

**4) Specific Points to Address:**

 - Consolidation: Reduce the number of figures and tables. For instance, some of the demographic data in Table 4 could be summarized in the text to maintain the "essay" flow.

 - Critical Debate: Enhance the "Introduction" by presenting a sharper debate on the conflict between "authenticity" and "commercialization" in LFT.

 - Formatting: Ensure all citations follow a consistent academic style and that the five-section structure is strictly followed without unnecessary subheadings.

 In conclusion, the article is technically robust and provides valuable empirical data for the field of business behavioral science. By adopting a more sophisticated narrative style and integrating the requested contemporary literature, the authors will significantly enhance the manuscript's academic contribution and originality. Addressing these points is essential for the article to be considered scientifically sound for final approval.

**References:**

 Phanpanya, K., Chanchaipitiphat, N., Jaepho, S., & Darmawijaya, I. G. (2025). From Niche to Necessity: A Multidimensional Evolution of Culinary Tourism and Post-Pandemic Resilience (2001-2025). Thai Arts and Culture Review, 1(2), Article 1. https://doi.org/10.14456/tacr.2025.6

 Walailak, P., Kajornatthapol, P., & Veerapong, S. (2025). Drivers of Customer Satisfaction in Northern Thailand’s Hotel Industry: The Marketing Mix and Service Quality Factors. Asian Administration and Management Review, 8(2), Article 10. https://doi.org/10.14456/aamr.2025.35

 Worrachananun, M. (2025). Sustainable Food Tourism: Impacts, Opportunity, and Challenges in The Modern Era. Asian Administration and Management Review, 8(1), Article 3. https://doi.org/10.14456/aamr.2025.3

Is the work clearly and accurately presented and does it cite the current literature?

Partly

If applicable, is the statistical analysis and its interpretation appropriate?

Yes

Are all the source data underlying the results available to ensure full reproducibility?

Yes

Is the study design appropriate and is the work technically sound?

Yes

Are the conclusions drawn adequately supported by the results?

Yes

Are sufficient details of methods and analysis provided to allow replication by others?

Yes

Reviewer Expertise:

Political Science, Public & Private Management, International Political Economy, Social Research

I confirm that I have read this submission and believe that I have an appropriate level of expertise to confirm that it is of an acceptable scientific standard, however I have significant reservations, as outlined above.
